# Nuclear segmentation facilitates neutrophil migration

**DOI:** 10.1242/jcs.260768

**Published:** 2023-06-08

**Authors:** Connie Shen, Eva Mulder, Wiebe Buitenwerf, Jérémy Postat, Aron Jansen, Matthijs Kox, Judith N. Mandl, Nienke Vrisekoop

**Affiliations:** ^1^ Department of Microbiology and Immunology, McGill University, Montreal, H3A 2B4, Canada; ^2^ McGill Research Centre for Complex Traits, McGill University, Montreal, H3G 0B1, Canada; ^3^ Department of Respiratory Medicine, Center of Translational Immunology, University Medical Center Utrecht, 3584 EA, Utrecht, The Netherlands; ^4^ Department of Physiology, McGill University, Montreal, H3G 1Y6, Canada; ^5^ Department of Intensive Care, Radboud University Medical Center, Nijmegen, 6525 GA, The Netherlands; ^6^ Radboud Center for Infectious Diseases (RCI), Radboud University Medical Center, Nijmegen, 6525 GA, The Netherlands

**Keywords:** Endotoxemia, Microfluidics, Migration, Neutrophil, Nuclear segmentation, Nucleus

## Abstract

Neutrophils are among the fastest-moving immune cells. Their speed is critical to their function as ‘first responder’ cells at sites of damage or infection, and it has been postulated that the unique segmented nucleus of neutrophils functions to assist their rapid migration. Here, we tested this hypothesis by imaging primary human neutrophils traversing narrow channels in custom-designed microfluidic devices. Individuals were given an intravenous low dose of endotoxin to elicit recruitment of neutrophils into the blood with a high diversity of nuclear phenotypes, ranging from hypo- to hyper-segmented. Both by cell sorting of neutrophils from the blood using markers that correlate with lobularity and by directly quantifying the migration of neutrophils with distinct lobe numbers, we found that neutrophils with one or two nuclear lobes were significantly slower to traverse narrower channels, compared to neutrophils with more than two nuclear lobes. Thus, our data show that nuclear segmentation in primary human neutrophils provides a speed advantage during migration through confined spaces.

## Teaser

**Highlighted Article:** Using heterogenous neutrophils mobilized following endotoxin administration in humans, we show that neutrophils with nuclei segmented into more lobes can migrate faster in a physically confined space.

## INTRODUCTION

Neutrophils are rapidly mobilized from the bone marrow into the blood in response to infection or injury, and are usually the first leukocytes to arrive in tissues, extravasating from blood vessels and navigating diverse microenvironments to reach affected sites. Using an amoeboid migration mode defined by low traction forces and a lack of focal adhesions (Kameritsch and Renkawitz, 2020; Yamada and Sixt, 2019), neutrophils achieve speeds of up to 30 μm/min, severalfold faster than other immune cells (Friedl and Weigelin, 2008; Manley et al., 2018). Leukocyte mobility within confined spaces such as tissues requires frequent shape changes and is a tightly coordinated cytoskeletal process, with actin retrograde flow generating forward motion in concert with myosin motors and microtubule networks (Lämmermann et al., 2008; Moreau et al., 2018). An important impediment to immune cells passing through tissue structure-imposed obstacles is the nucleus, the largest and most rigid organelle in a cell. The relative stiffness of the nucleus, which is determined largely by the composition of the nuclear envelope lamina and the degree of chromatin condensation, can thus hamper the ability of cells to move quickly in complex environments. Among nuclear lamina proteins, lamin A/C (encoded by *LMNA*) is regarded as a key determinant of nucleus stiffness, and compared to non-haematopoietic cells, fast-moving immune cells such as T cells and neutrophils in particular express lamin A/C at very low levels (Harada et al., 2014; Wolf et al., 2013). In some instances, the physical deformation of the nucleus that occurs during the passage through small pores, such as those in basement membranes or collagen-dense skin, can even lead to nuclear envelope rupture (Davidson et al., 2014; Friedl et al., 2011; Salvermoser et al., 2018; Thiam et al., 2016). Additionally, recent work showing that the nucleus can act as a size gauge for migratory path selection (Renkawitz et al., 2019) underscores that the bulky nucleus presents a challenge for mobile cells, particularly when cells must move rapidly.

One key feature that differentiates the neutrophil nucleus from that of other leukocytes is its unique shape. In human neutrophils, the nucleus can range from having a ‘banded’ horseshoe-like shape to being hypersegmented with five or more lobes. This nuclear segmentation is a feature common to both humans and mice; however, in murine neutrophils the nucleus assumes a circular configuration, whereas in human neutrophils the nucleus adopts a linear configuration (Carvalho et al., 2015). The current paradigm is that the smaller nuclear diameter and reduced steric hinderance of the multilobular ‘pearls-on-a-string’ arrangement allows for greater cell flexibility and thus enables faster migration, particularly through tight spaces (Liew and Kubes, 2019; Manley et al., 2018; Wolf et al., 2013). However, there currently exists only limited experimental evidence for this enduring hypothesis. Previous studies have demonstrated that human neutrophils can pass through smaller pores and migrate through dense collagen matrices with greater speed compared to tumour cells or even T cells (Wolf et al., 2013). Work directly investigating the role of nuclear segmentation itself in neutrophil migration has relied on manipulating nuclear envelope composition. In one notable study, expression of lamin A/C and lamin B receptor (LBR) was altered in neutrophil-differentiated HL-60 cells *in vitro* to obtain neutrophils retaining a circular nucleus, mimicking the lamin A/C downregulation and LBR upregulation that is necessary for nuclear segmentation during neutrophil development (Rowat et al., 2013). Ultimately, the authors of this previous study found that the multilobed nuclear shape is not necessary for passage through 5 μm constrictions or 3 μm Transwell pores (Rowat et al., 2013). Similarly, in clinical observations of Pelger–Huët anomaly (PHA), a genetic disorder defined by mutations in LBR, the hypolobulated neutrophils from people with PHA do not show clear impairments in cell movement and chemotaxis (Speeckaert et al., 2009). However, an important caveat of these studies is that nuclear envelope proteins have physiological roles that might impact migration directly, or indirectly through modified gene regulation, beyond their effects on nucleus lobularity (Davidson and Lammerding, 2014; Gaines et al., 2008; Szymczak et al., 2022). Furthermore, the *in vitro* differentiated HL-60 immortalized cells differ from peripheral blood neutrophils in their nuclear composition, including in lamina content and heterochromatin density (Olins et al., 2008).

Here, we sought to determine whether nuclear lobulation facilitates the ability of neutrophils to migrate in small spaces in a physiological setting, comparing the migration of human neutrophils with varying degrees of nuclear segmentation in microfluidic devices. During homeostasis, circulating neutrophils are a relatively homogeneous population of matured, differentiated cells with two to four nuclear lobes (Liew and Kubes, 2019). However, in response to inflammatory stimuli such as endotoxin, an additional pool of neutrophils is rapidly recruited to the circulation, and neutrophils released into the blood span a wide range of nuclear phenotypes ranging from banded to hypersegmented (Manz and Boettcher, 2014). We obtained circulating neutrophils from individuals given a low dose of endotoxin to induce a controlled emergency granulopoiesis response, and we leveraged the increased diversity in neutrophil nuclear lobularity to directly address the question of whether nuclear phenotype influences migratory capacity. We found that greater lobularity led to increased cell velocity along the chemoattractant gradient when neutrophils migrated through narrow paths.

## RESULTS AND DISCUSSION

### Neutrophil subsets display differential migratory behaviours correlating with nuclear lobularity

**Fig. 1.**
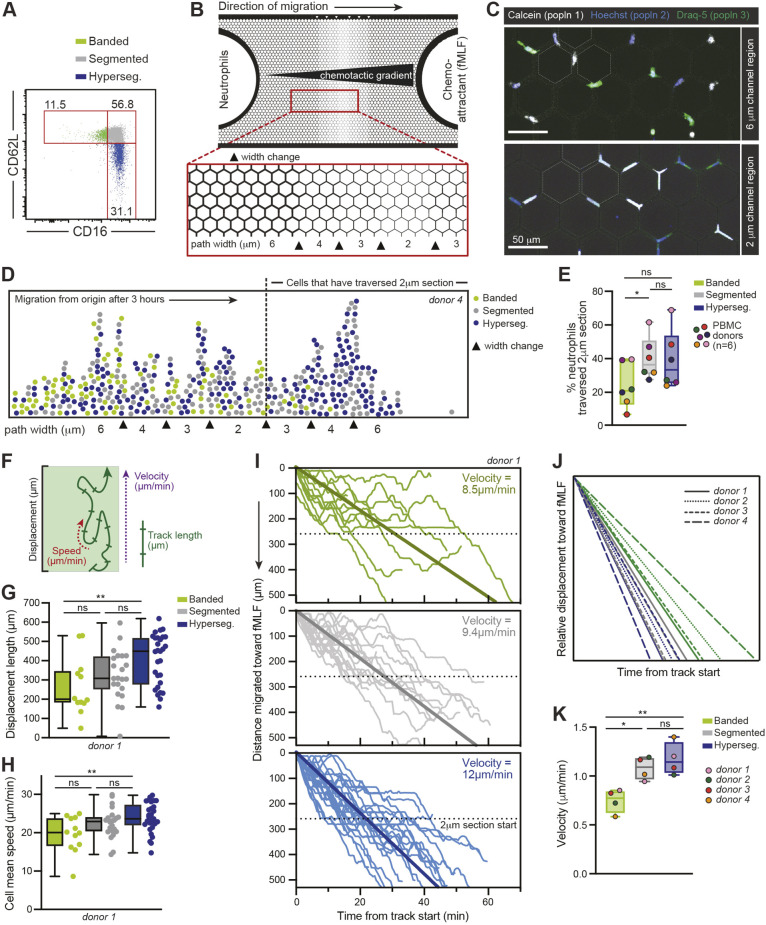
**Neutrophil subsets display differential ability to migrate through tight spaces.** (A) Representative FACS plot of CD16 and CD62L expression by neutrophils sorted from blood 3 h after low-dose endotoxin administration. Numbers indicate percent of cells in each gate. (B) Schematic of the custom-made pillar forest microfluidic device used in migration assays. Neutrophils were seeded on one side and 10^−7^M fMLF chemoattractant was added on the other side, such that cells migrated across the pillar forest in paths that decreased in width in a stepwise fashion from 6 μm to 4 μm to 3 μm to 2 μm along the chemoattractant gradient. (C) Example static images of three subsets of sorted neutrophils differentially stained with fluorescent dyes (Hoechst 33342, Calcein-AM or Draq-5). Dashed lines mark a subset of pillars to illustrate the difference in channel width in the two regions. Fluorescent neutrophils were visualized either by a static tile scan of the entire microchannel when at least 25% of the cells had passed through the 2 μm section (D,E) or by time-lapse microscopy (F–K). (D) Representative example from donor 4 of distance migrated by each neutrophil subset within the pillar forest after 3 h. Path width changes are indicated by arrowheads; dotted line represents the point at which cells have traversed the 2 μm section. *n*=251 cells. (E) Quantification of the percentage of cells that had crossed the 2 μm section after 3 h (as shown in D). Data are from *n*=6 human donors, 150–450 cells analysed per donor. *P*-values from one-way ANOVA with Tukey's multiple comparisons post hoc test are shown (ns, not significant; *P≤0.05). (F) Schematic illustrating the measured cell migration parameters: track length, speed, displacement and velocity. Track length is the total distance the cell has travelled. Speed is the rate of movement along the track. Displacement is the total distance travelled towards the chemoattractant. Velocity is the rate of movement in the direction of the chemoattractant source over the course of the cell track. (G–I) Representative plots of total displacement length (G) and cell mean speed (H), calculated by neutrophil subset for cells from donor 1 (total of *n*=63 cells). Individual data points for each subset are shown alongside the boxplots. *P*-values from one-way ANOVA with Tukey's multiple comparisons post hoc test are shown (ns, not significant; *P≤0.05). (I) Normalized tracks by subset (colour coded as in G,H) shown from one donor (donor 1), total of *n*=63 cells. Tracks are displayed as distance migrated towards the chemoattractant source as a function of time. Velocities were calculated as an average of the simple linear regression of each track. Bold lines show the average regression line across all tracks. Dotted lines indicate the start of the 2 μm section. Data in G–I are representative of four donors. (J,K) Relative velocities were calculated as in I for *n*=4 different donors, 40–65 cells analysed per donor. Velocities were normalized to the average velocity within each donor. Average velocities shown by subset (J; subsets colour coded as in G,H) and boxplots showing normalized velocity as calculated per donor (K). *P*-values from one-way ANOVA with Tukey's multiple comparisons post hoc test are shown (ns, not significant; *P≤0.05; **P≤0.01). Boxplots in E,G,H and K show the median (horizontal line), interquartile range (box) and range (whiskers). Hyperseg., hypersegmented; PBMC, peripheral blood mononuclear cell; popln, population.

We obtained circulating neutrophils from human donors given a low dose of endotoxin to investigate the limits of the ability of neutrophils with different nuclear segmentation phenotypes to migrate through tightly constricted spaces. During the endotoxemia response, neutrophils released into the blood can be sorted into subsets based on their CD16 (FcγRIII) and CD62L (L-selectin) expression. The homeostatic pool of CD16^high^ CD62L^high^ neutrophils has standard segmented nuclei (two to three lobes), the CD16^low^ subset is enriched for neutrophils with banded nuclei (one lobe) and the CD16^high^CD62L^low^ subset is enriched for hypersegmented nuclei (four or more lobes) (Grinsven et al., 2019). We used fluorescence-activated cell sorting (FACS) to sort neutrophils into these three subsets based on CD16 and CD62L expression, and we refer to these subsets as banded, segmented or hypersegmented hereafter (Fig. 1A). To observe the sorted neutrophils migrating in increasingly narrow spaces, we employed custom-fabricated microfluidic devices, which serve as a reductionist approach to studying complex three-dimensional migration dynamics and allow for the tracking of single-cell spatiotemporal patterns (Heuzé et al., 2011). The microfluidic devices were made of polydimethylsiloxane (PDMS) in a pillar-forest design, where the channels through which the cells migrated had a fixed height of 5 μm but reduced in width in a stepwise fashion across the device from 6 μm to 4 μm to 3 μm to 2 μm before increasing in width stepwise back up to 6 μm (Fig. 1B). The 6 μm width was considered to be fairly permissive, whereas the 2 μm width presented a significant challenge for cells to migrate through due to the channel width being smaller than the diameter of the nucleus (Thiam et al., 2016; Wolf et al., 2013). Neutrophils were seeded on one side of the microchannel, and the chemoattractant f-Met-Leu-Phe (fMLF) was added on the other side to diffuse through the microchannels, creating a chemotactic gradient. The three sorted subsets of neutrophils were stained with distinct fluorescent dyes (Hoechst 33342, Calcein-AM or Draq-5), mixed at equal ratios and added to our fabricated pillar forest. Dye labels were rotated between different donors, and we confirmed that the dyes did not differentially affect migration speed by dye labelling neutrophils from non-lipopolysaccharide (LPS)-treated donors (controls) and measuring cell speed (Fig. S1A). As the neutrophils traversed from the region of 6 μm-wide channels (hereafter referred to as the 6 μm section) to the region of 2 μm-wide channels (hereafter referred to as the 2 μm section), the cells and their nuclei became increasingly constrained and elongated (Fig. 1C). Of note, we have previously shown that the process of transmigration through small pores does not itself induce nuclear segmentation in neutrophils (Grinsven et al., 2019).

Next, we assessed differences between neutrophil subsets in their ability to move within the more constrained channels using two approaches: by taking a static image after 3 h of migration in the microfluidic device (Fig. 1D,E), and by dynamic imaging of cell behaviour over time (Fig. 1F–I). Given that the 2 μm section was the most challenging region for migrating cells to navigate through, we first quantified the total number of neutrophils that were able to successfully pass through this whole section as proxy for migration capacity. Compared to the segmented and hypersegmented subsets, a smaller proportion of the banded neutrophil subset crossed the 2 μm section in 3 h, even though the subsets were present in similar proportions prior to reaching the 2 μm section (Fig. 1D). Performing this analysis across six donors, we found a robust difference between the banded subset and the segmented and hypersegmented subsets in the percentage of neutrophils that traversed the 2 μm section (Fig. 1E). Second, we performed live-cell imaging of the dynamic behaviour of neutrophils migrating through the channels (Movie 1). From our videos, we tracked individual cell behaviours by subset, including track length, speed, displacement and velocity. We defined the track length as the total distance travelled by the cell, the mean speed as the average rate of movement along the track, the displacement as the total distance travelled towards the chemoattractant source and the velocity as the rate of movement in the direction of the chemoattractant over the course of the cell track (Fig. 1F). Overall, the banded subset had significantly lower mean cell speed and displacement compared to the hypersegmented subset of neutrophils (Fig. 1G,H), without a reduction in track duration, length or straightness (Fig. S1B). Analyzing the migration distance towards the chemoattractant as a function of time for each cell, we observed that there were significant differences in the speed at which each subset could traverse the pillar forest. The average velocities for the banded, segmented and hypersegmented subsets were 8.5, 9.4 and 12 μm/min, respectively (Fig. 1I). Furthermore, the reduced speeds observed in the banded subset could be attributed to the narrower paths, since we observed similar speeds for all three subsets in the wider 6 μm section (Fig. S1B). Although there was donor-to-donor variation in the average neutrophil cell speed, relative velocities amongst the neutrophil subsets nonetheless robustly demonstrated that the banded subset of neutrophils had a migratory disadvantage compared to the other subsets (Fig. 1J,K). Based on a previously published proteomics dataset (Tak et al., 2017), these differences were not explained by FPR1, LMNB1, LMNB2 or LBR expression, which did not differ significantly in protein expression levels between subsets (Fig. S1C,D).

### Neutrophils with single-lobed nuclei have a reduced capacity to migrate through narrow channels

**Fig. 2.**
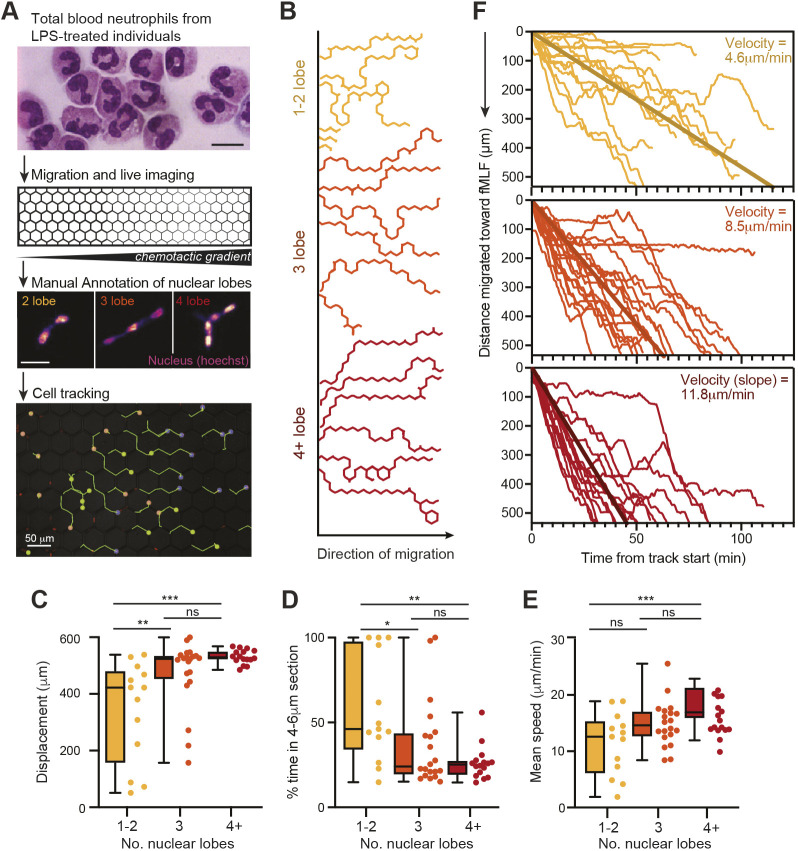
**Greater nuclear lobularity in neutrophils confers increased migratory capacity.** (A) Schematic of workflow. After 3 h of intravenous endotoxin administration, total blood neutrophils were stained with nuclear dye Hoechst 33342, run through the pillar forest migration assay, and cells were visualized by time-lapse microscopy. Videos were analysed by cell tracking and manual nucleus lobule annotation. Neutrophils were annotated as having either one or two, three, or four or more lobes. Example annotations and tracking data are shown. Scale bars: 10 μm unless indicated otherwise. (B) Representative example of cell tracks, as relative *x* and *y* coordinates, shown by nucleus lobularity group. (C–E) Total displacement length (C), percentage of time spent in the 4 μm and 6 μm sections (D) and cell mean speed (E), calculated by nucleus lobularity group. *n*=49 cells in total from one donor. Individual data points for each subset are shown alongside the boxplots. Boxplots show the median (horizontal line), interquartile range (box) and range (whiskers). *P*-values from one-way ANOVA with Tukey's multiple comparisons post hoc test are shown (ns, not significant; *P≤0.05; **P≤0.01; ***P≤0.001. (F) Normalized tracks by subset shown from one donor, *n*=49 cells. All tracks are shown, including those also shown in B. Tracks are displayed as distance migrated towards the chemoattractant source as a function of time and are colour coded as in B. Velocities were calculated as an average simple linear regression of each track. Bold lines show the average regression lines across all tracks. Data in C–F are representative of three donors (45–60 cells per donor).

One caveat of utilizing CD16 and CD62L as markers for neutrophil nucleus segmentation is that there is a substantial overlap in nucleus lobularity between subsets, even if the average number of nuclear lobes differs (Fig. S1E). Moreover, there are other functional differences described between the subsets, including gene expression differences, that could impact cell migration (Pillay et al., 2010, 2012; Tak et al., 2017). Therefore, we next investigated the relationship between the number of nucleus lobes and neutrophil migration more directly. Instead of sorting subsets based on surface marker expression, we labelled total blood neutrophils from endotoxin-treated donors with the fluorescent nuclear dye Hoechst 33342 and performed the same microchannel migration assay as before, manually annotating cells according to their number of nuclear lobes (Fig. 2A). We were able to robustly discern neutrophils with one or two, three, and four or more nuclear lobes (Fig. 2). Once annotated, cell movements were tracked (Movie 2), and the migratory tracks were compared (Fig. 2B). Our data revealed that neutrophils with one or two nuclear lobes were less able to traverse the smallest 2 μm section, as shown by the significantly reduced track displacement, greater percentage of time spent in the 4 μm and 6 μm sections, and reduced mean speed (11.3 μm/min) compared to that of neutrophils with three nuclear lobes or with four or more nuclear lobes, which migrated at average speeds of 14.9 μm/min and 17.9 μm/min, respectively (Fig. 2C–E). Thus, neutrophils with more segmented nuclei not only travelled faster, but also moved a greater distance despite having similar total track duration and track length (Fig. S2A–C). Indeed, analyzing neutrophil migration distance towards the chemoattractant as a function of time, we observed that neutrophils with three nuclear lobes or with four or more nuclear lobes migrated across the channel with greater velocity towards the chemoattractant source (8.4 μm/min and 11.8 μm/min, respectively) than the neutrophils with one or two nuclear lobes (4.6 μm/min) (Fig. 2F). Overall, our results showed that the variation in migratory speeds observed among neutrophils could be explained by the extent of their nuclear segmentation, and this relationship was more pronounced when grouping neutrophils strictly by their nuclear lobularity rather than by phenotype-defined subset.

Our study of the migratory behaviour of unmanipulated human neutrophils provides evidence supporting the theory that nuclear segmentation facilitates cell navigation of tight spaces. We have previously reported that non-segmented banded neutrophils are not restrained in three-dimensional collagen matrices (Grinsven et al., 2019). However, these complex matrices have variable pore sizes (Wolf et al., 2013), and neutrophils have been shown to probe for the widest path (Renkawitz et al., 2019). Here, we precisely defined the environmental constraints imposed on neutrophils using custom-designed microfluidic devices to prevent them from choosing the widest path and to force them into ever-tighter channels. In capturing the dynamics of individual neutrophils migrating through decreasing path widths, we observed that banded neutrophils were less able to traverse the smallest 2 μm section than segmented and hypersegmented neutrophils. Moreover, we showed that the effect of nuclear lobularity on migratory behaviour is greater than that of neutrophil subtype defined by CD16 and CD62L expression. This supports the conclusion that neutrophil lobularity, rather than their functional subset, plays a greater role in determining migration efficiency in this context. However, there might be gene expression changes that track with lobulation that we have not examined in the present study, and we cannot rule out a role for an unidentified factor correlating with nuclear lobularity that affects neutrophil migratory differences. Nonetheless, our work gives credence to the idea that nuclear shape in neutrophils is an evolutionary adaptation allowing better navigation through narrow pores, enabling neutrophils to function as ‘first responders’ in tissue upon insult or injury.

In cells containing classically round nuclei, this large rigid organelle is a hindrance to migration. Cells passing through small constrictions can experience nuclear blebbing, lamina rupture and nuclear envelope rupture (Denais et al., 2016; Raab et al., 2016; Srivastava et al., 2021; Thiam et al., 2016). If this is the case, the reduction of intranuclear pressure as well as lesser steric hinderance when the nucleus is segmented might explain the ability of neutrophils with greater number of nuclear lobes to pass through tighter constrictions with relative ease. It has also been reported that the neutrophil nucleus, rather than deforming, can unfold to migrate through tight pores (Wolf et al., 2013). Future investigations into the biophysical properties of differentially segmented neutrophil nuclei, such as rigidity and flexibility, will better define the mechanism by which neutrophils migrate with such efficiency.

The notion that neutrophils are a short-lived, homogeneous population has been increasingly challenged in studies of neutrophils at homeostasis and in various pathological states such as infection and cancer (Bruijnzeel et al., 2015; Hellebrekers et al., 2018; Ng et al., 2019; Quail et al., 2022; Silvestre-Roig et al., 2019; Voisin and Nourshargh, 2019; Xie et al., 2020). Indeed, the presence of neutrophils with different functional capabilities can have significant effects on disease outcome. Our data suggest that an important variable in characterizing neutrophil heterogeneity with regard to their migratory behaviour is the extent of nuclear lobulation. It will be interesting to investigate whether there are differences among neutrophils with regard to arrival time at a site of local tissue injury and infection, with hypersegmented neutrophils reaching sites in denser tissue first, and their subsequent inflammatory response rendering tissue more permissive for banded neutrophils and other mono-lobed leukocytes to infiltrate.

## MATERIALS AND METHODS

### Human blood samples

For volunteers treated with endotoxin, blood samples were acquired from a random 17 out of 100 volunteers who participated in the 100LPS human endotoxemia study (NL68166.091.18, CMO: 2018-4983). All volunteers signed a written informed consent form. The study was approved by the ethics review board of the Radboud University Medical Center. Participants were both male and female, aged 18–35, and were healthy as confirmed by physical examination, electrocardiography, medical history and multiple laboratory tests. Among the exclusion criteria were pregnancy, smoking and recent hospital admission.

On the day of the LPS challenge, volunteers were hospitalized at the Radboud University Medical Center and received a single intravenous administration of 1 ng/kg LPS (Escherichia coli O:113; List Biological Laboratories, Campbell, CA, USA), inducing controlled systemic inflammation. Participants were constantly monitored by a care physician for sepsis-related symptoms such as high blood pressure, high heart rate or fever, among others. Blood samples were collected 3 h after LPS administration. For non-endotoxin-treated controls, human blood samples were obtained from healthy volunteers, both male and female, aged 18–65. All donors signed an informed consent form, and sampling was approved by the Biobanks Review Committee of the University Medical Center Utrecht (approval code 18/774, approval date 25 June 2013). All clinical investigations were conducted according to the principles expressed in the Declaration of Helsinki.

### Neutrophil isolation

Blood samples were collected in sodium heparin tubes (BD Vacutainer). Cold (4°C)-shock buffer (0.1 mM Na_2_EDTA, 10 mM KHCO_3_ and 150 mM NH_4_Cl in double distilled water with pH adjusted to 7.4) was added to the blood to lyse erythrocytes. Next, white blood cells were washed once with PBS2+ [0.32% sodium citrate and 4 g/l human albumin (Sanquin, Amsterdam, The Netherlands) in phosphate-buffered saline] and stained with antibodies against CD16 (Beckman Coulter, 3G8) and CD62L (Biolegend, DREG-56) at 1:100 dilution. After staining, neutrophil subsets were sorted using a BD FACSAria TM 3 Cell sorter (BD). First, singlets were selected based on forward scatter height (FSC-H) and forward scatter area (FSC-A). Neutrophils were gated by forward and side scatter area, whereafter neutrophil subsets were sorted on differential CD16 and CD62L expression levels (Fig. 1). Sorted subsets were acquired in FACS tubes containing PBS2+ buffer. For experiments using total neutrophils, these were isolated from blood by Ficoll density separation. In short, whole blood samples from healthy controls were diluted 1:1 in PBS2+ and layered on Ficoll-Paque Plus (GE Healthcare), followed by the red blood cell-shocking procedure described above. Cells were washed with HEPES3+ buffer comprising 20 mM HEPES, 132 mM NaCl, 6 mM KCl, 1 mM MgSO_4_, 1.2 mM KH_2_PO_4_, 1.0 mM CaCl_2_, 5 mM glucose and 5 mg/ml human serum albumin (Albuman 200 g/l; Sanquin, Amsterdam, The Netherlands) at a pH adjusted to 7.4. This procedure yielded a purity of >90% neutrophils. Isolated neutrophils were stained with either Hoechst 33342 (AnaSpec; 4 μM), Calcein-AM (Molecular probes; 0.25 μM) or Draq-5 (eBioscience; 20 μM).

### Cytospins and nuclear morphology

Neutrophils were seeded on a standard microscope slide (Menzel Gläser, Thermo Fisher Scientific) using a cytocentrifuge (Shandon cytospin 2, Block Scientific). May–Grünwald (Merck) and Giemsa (Merck) staining was applied, and images were obtained with an Axioskop 40 microscope (Zeiss) using a 100× oil immersion objective objective. Lobes were classified as separate when the connection between two adjacent lobes was smaller than a third of the width of the adjacent nucleus.

### Microfluidic channels

Microfluidic devices were prepared as previously described (Huezé, 2011). Briefly, PDMS (Momentive Performance Materials, RTV615) was poured into our previously manufactured custom-design moulds (4D Cell). Air bubbles were removed by vacuum chamber then incubated for 1 h at 100°C or 24 h at room temperature. PDMS microfluidic devices were removed from the moulds with isopropanol and cleaned with ethanol. The devices were plasma cleaned on high intensity for 2 min (Harrick Plasma). PDMS moulds were then irreversibly bound to a glass-bottomed dish (WPI FluoroDish). Prior to use, microchannels were plasma cleaned on high intensity for 3 min. Channels were coated with 10% human albumin (200 g/l; Sanquin, Amsterdam, The Netherlands) in PBS for 1 h at 37°C, 5% CO_2_. Thereafter, channels were incubated with HEPES3+ buffer. Between coating and incubation steps, wells were washed with PBS2+.

### Migration assays and microscope image acquisition

Neutrophils with different fluorescent labelling (Hoechst 33342, Calcein-AM or Draq-5, as described above) were mixed together in a 1:1:1 ratio with a final concentration of 1×10^8^ neutrophils/ml then loaded into the seeding well of the microfluidic channel. The well on the other side of the microfluidic channel was loaded with 10^−7^M fMLF chemoattractant (Sigma-Aldrich). Microfluidic channels were imaged using a confocal microscope (Zeiss, LSM710) or a fluorescence microscope (Olympus, IX83). Time-lapse images of one focal plane were obtained using a 20× air objective with a 30 s interval per timepoint. Microfluidic channels were maintained at 37°C. To exclude the possibility of staining dyes influencing neutrophil migration, the dyes were rotated for the three subsets across experiments.

### Analysis

Static images were processed and quantified using Fiji (ImageJ). Time lapses were processed and analysed with Imaris (version 9.1; Oxford Instruments) and Python (version 3.8). Data was graphed and statistics performed using Prism 8 (Graphpad). *P*<0.05 was considered significant, and tests used are specified in figure legends.

## Supplementary Material

10.1242/joces.260768_sup1 Supplementary information
