# Supplementary material for: Nuclear segmentation facilitates neutrophil migration
Source: J Cell Sci. 2023 Jun 8;136(11):jcs260768. doi: 10.1242/jcs.260768 (PMC10309577; doi:10.1242/jcs.260768)
Supplement: Supplementary information [file joces-136-260768-s1.pdf]

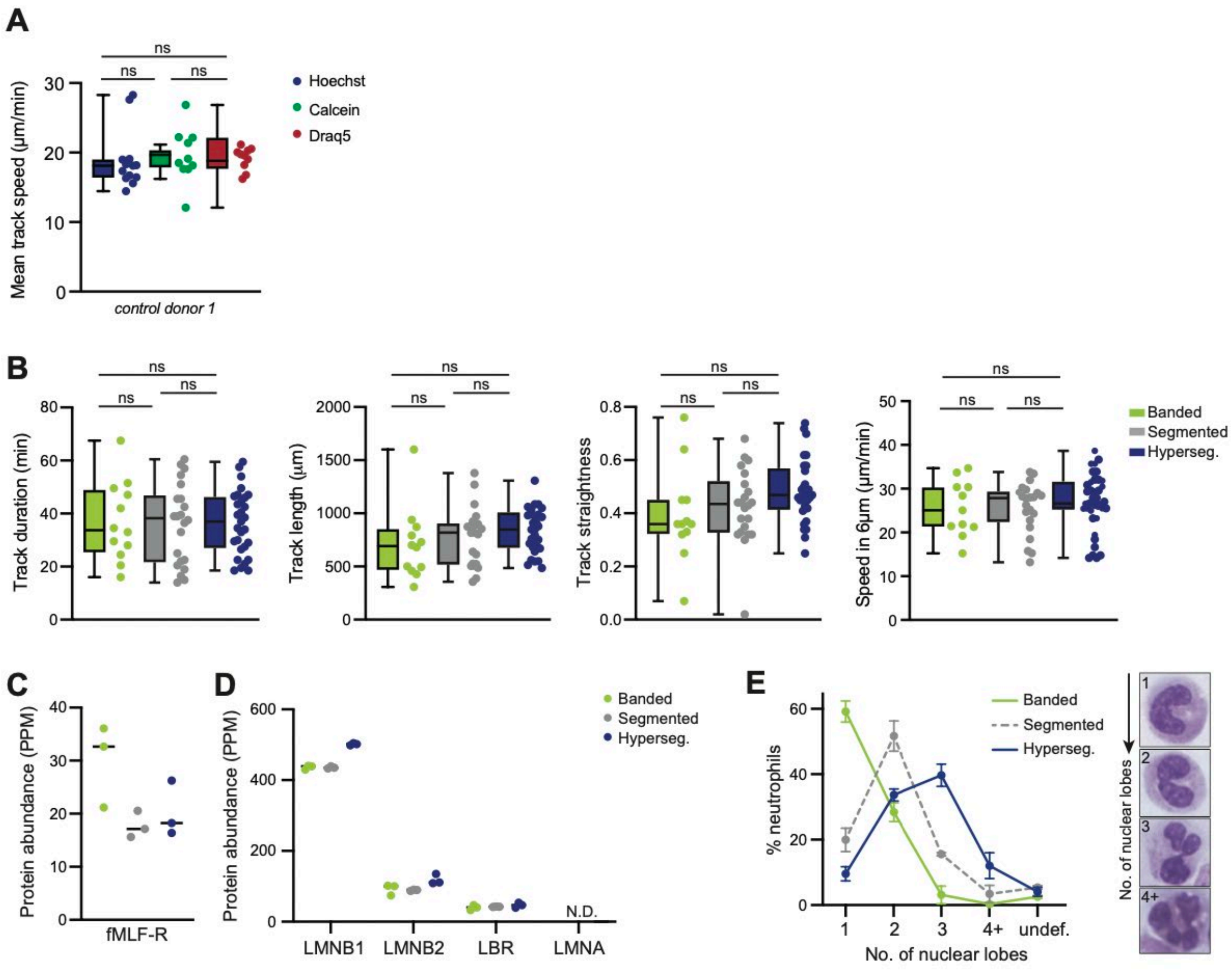

Journal of Cell Science • Supplementary information

Fig. S1. Differences in chemokine receptor expression, nuclear lamina composition and dye-labeling does not explain migration differences between neutrophil subsets.
(A) Total blood neutrophils from a non-endotoxin treated donor were isolated and differentially stained with fluorescent dyes (Hoechst 33342, Calcein-AM, or Draq-5). Mean track speeds are shown from n = 33 cells (**B**) Track duration, track length, track straightness, and cell speed in the 6μm section quantified by neutrophil subset for n=63 cells from one donor. Track duration is the total amount of time the cell is tracked; track length is the total distance the cell has travelled over the course of the track; track straightness is a calculation of the cell displacement / track length; cell speed in the 6μm section is the mean speed at the beginning of each track. P values from one-way ANOVA are shown; $*P \leq 0.05$. **(C, D)** Proteomics of FACS-sorted neutrophils based on CD16 and CD62L expression from previously published dataset provided at ProteomeXchange Consortium via the PRIDE partner repository (data set identifier PXD001674; DOI: 10.6019/PXD001674). Protein levels for fMLF receptor (C), Lamin B1 (LMNB1), Lamin B2 (LMNB2), Lamin B receptor (LBR), and LaminA/C (D) are shown. N.D., not detectable. **(E)** Quantification of number of lobes per neutrophil in CD16/CD62L-expression sorted subsets from cytospins of May-Grünwald and Giemsa stained cells. Data is quantified from 3 donors, 100 cells per donor. Values shown are median and range.

Journal of Cell Science • Supplementary information

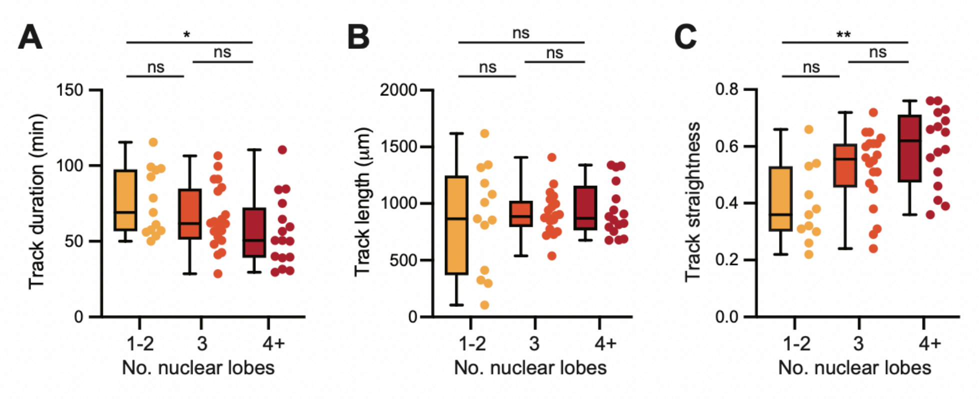

Fig. S2. Cell track parameters in neutrophils with different nuclear lobe numbers.

(A) Track duration (total amount of time the cell is tracked), (**B**) track length (the total distance the cell has travelled over the course of the track), and (**C**) track straightness (cell displacement / track length) shown by nucleus lobularity group, n=49 cells. P values from one-way ANOVA are shown; *$P \leq 0.05$, **$P \leq 0.01$, ***$P \leq 0.001$.

Journal of Cell Science • Supplementary information

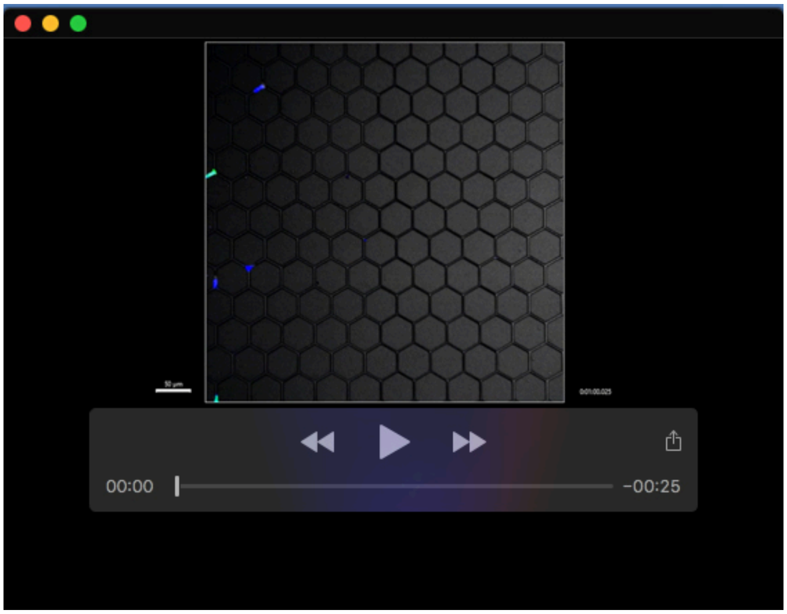

Movie 1. Time lapse video of differentially stained neutrophils, related to Figure 1. Part A shows cells only, and Part B shows coded cell tracks.

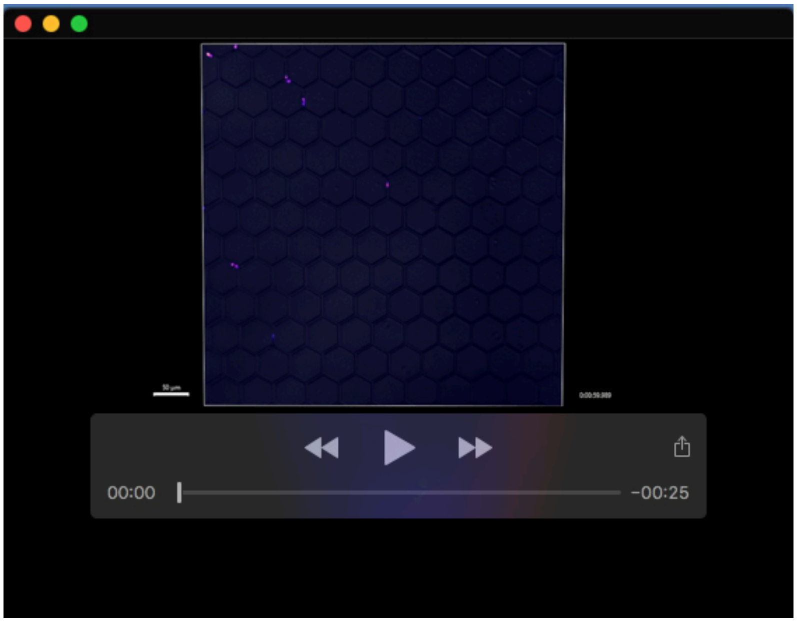

Movie 2. Time lapse video of Hoechst-stained neutrophils, related to Figure 2. Part A shows cells only, and Part B shows coded cell tracks.

Journal of Cell Science • Supplementary information
