# Supplementary material for: Nuclear segmentation facilitates neutrophil migration
Source: J Cell Sci. 2023 Jun 8;136(11):jcs260768. doi: 10.1242/jcs.260768 (PMC10309577; doi:10.1242/jcs.260768)
Supplement: Supplementary file 1 [file joces-136-260768_review_history.pdf]

Nuclear segmentation facilitates neutrophil migration

Connie Shen, Eva Mulder, Wiebe Buitenwerf, Jérémy Postat, Aron Jansen, Matthjis Kox,
Judith N. Mandl and Nienke Vrisekoop
DOI: 10.1242/jcs.260768

Editor: Daniel Billadeau

Review timeline

Original submission:	31 October 2022
Editorial decision:	16 December 2022
First revision received:	16 March 2023
Editorial decision:	17 April 2023
Second revision received:	1 May 2023
Accepted:	3 May 2023

Original submission

First decision letter

MS ID#: JOCES/2022/260768

MS TITLE: Nuclear segmentation facilitates neutrophil migration

AUTHORS: Judith N Mandl, Nienke Vrisekoop, Connie Shen, Eva Mulder, Wiebe Buitenwerf, Aron Jansen, Matthjis Kox, and Jeremy Postat
ARTICLE TYPE: Short Report

We have now reached a decision on the above manuscript.

To see the reviewers' reports and a copy of this decision letter, please go to: https://submit-jcs.biologists.org and click on the 'Manuscripts with Decisions' queue in the Author Area. (Corresponding author only has access to reviews.)

As you will see, the reviewers raise a number of substantial criticisms that prevent me from accepting the paper at this stage. They suggest, however, that a revised version might prove acceptable, if you can address their concerns. If you think that you can deal satisfactorily with the criticisms on revision, I would be pleased to see a revised manuscript. We would then return it to the reviewers.

Please ensure that you clearly highlight all changes made in the revised manuscript. Please avoid using 'Tracked changes' in Word files as these are lost in PDF conversion.

I should be grateful if you would also provide a point-by-point response detailing how you have dealt with the points raised by the reviewers in the 'Response to Reviewers' box. Please attend to all of the reviewers' comments. If you do not agree with any of their criticisms or suggestions please explain clearly why this is so.

© 2023. Published by The Company of Biologists under the terms of the Creative Commons Attribution License (https://creativecommons.org/licenses/by/4.0/).

Reviewer 1

Advance summary and potential significance to field

The authors used a low level of LPS to induce neutrophils with different maturation stages and numbers of nuclear lobes into circulation. They then isolated the neutrophils, sorted them based on the surface marker, and let them migrate in microfluidic with increasingly narrower spaces. They also stained the nucleus and manually counted the lobs. They discovered a significant correlation between the number of lobes with the neutrophil's ability to migrate in narrow spaces. This is the first report showing this correlation, which is in contrast to previous experiments using other settings. Therefore the results are novel and of interest to the cell migration field.

Comments for the author

The limitation of the current manuscript is a lack of mechanistic understanding.
Does the number of lobes correlate with a softer or more flexible nucleus?
In addition, the nu mber of lobes is a direct indication of the maturation stages of neutrophils. The result can be interpreted alternatively that mature neutrophils have an increased ability to migrate through confined spaces. In addition to the morphology of the nucleus, there are significant gene expression changes as neutrophils mature.
This is a significant limitation of the current study, and I feel the manuscript is not suitable for publication in JCS.

Reviewer 2

Advance summary and potential significance to field

The authors have submitted a manuscript describing the effect of nuclear lobulation on migration through narrow microfluidic channels in heterogeneous populations of primary human neutrophils, demonstrating that neutrophils with more lobes had an improved ability to migrate through the narrow channels than neutrophils with less lobulated nuclei. The manuscript uses ingenious microfluidic networks to study the migration of the cells in confined conditions and relates the migration behavior to various molecular and morphological parameters, identifying nuclear lobulation as a key determinant for confined migration. The movies included in the manuscript are beautiful to watch, and the fact that the authors used primary human fibroblasts and highlight the heterogeneous nature of the cells is considered a major strength.

Comments for the author

Nonetheless, the manuscript in its current form has several limitations that must be addressed by the authors to better support their interpretation, provide context for the findings, and improve the robustness of the findings.

1) In their presentation of the results and their interpretation, the authors refer to previous work characterizing human neutrophils, particularly their own prior work to describe the association between nuclear lobulation and C16 and CD62L levels (Ginsven et al. J Immunology 2019) and a previous proteomic study (Tak et al. Blood 2017). Since that the authors work with primary human cells, which may be extremely variable, it is important to confirm the results of the prior studies in their current experimental system, especially when correlating results from their current measurements with data from those previous studies to investigate a potential association between protein levels vs. nuclear morphology (Supp. Fig. 1C-D). The authors should provide data for protein measurements in the same cells as used in their experiments, rather than simply assume that the cells used in their studies have similar composition as reported previously. If the data presented in Figure S1C-D is from new measurements, rather than literature data, the authors should clarify this in the manuscript and provide a description of the measurements in the Methods section. For the results presented in Fig. S1E, the authors should relate the number of nuclear lobes to the levels of CD16 and CD62L, i.e., the classification they used in the current manuscript, and not just as function of the three different categories ‘banded’, ‘mature’, and ‘hypersegmented’). If these categories are considered to be equivalent, this should be explicitly stated and validated.

© 2023. Published by The Company of Biologists under the terms of the Creative Commons Attribution License (https://creativecommons.org/licenses/by/4.0/).

2) Although the authors' results convincingly demonstrate that neutrophils with highly lobulated nuclei pass through the channel network more efficiently, the current analysis does not sufficiently address if the effect is specific to the migration through very narrow channels, and if improved chemotaxis by the highly lobulated cells could contribute to the effect. To answer these questions, the authors should calculate migration speed for the different categories of neutrophils as a function of the channel size (i.e., do the highly lobulated cells also show a higher migration efficiency in the larger channels, or only in the narrower channels) and also test whether the same effect is observed in the absence of a chemoattractant or when performing chemotaxis experiments in larger channels that do not require nuclear deformation.

3) The current description of the migration analysis is difficult to follow due to the confusing terminology and missing definition of terms. For example, the authors use "speed" to describe the rate of movement of the cells along their path (which is consistent with the physical definition of speed), but use "velocity" (which is normally defined as the vector description of the rate and direction of an object's movement) as the directional rate of movement of the cells towards the chemoattractant, if this reviewer's interpretation of the schematic in Figure 1F is correct. Furthermore, the authors refer to "track length, track length, speed, and displacement" and "track duration, length or straightness" in the text, without ever adequately defining these terms. Without a better definition of these terms, the results are very difficult to follow and interpret. The authors should provide a comprehensive and more detailed definition of all the terms used to characterize the cell movement through the microfluidic channels, as these definitions are critical to assess how the nuclear lobulation is association with difference in absolute migration speed, directionality, persistence, chemotaxis, and other parameters of migration.

4) Some figures legends are missing important information on the number of cells used per donor, such as Figures 1E-K. The results presented in panels 1G-I appear to be from a single donor. Were similar results in cells from additional donors?

5) The discussion of previous studies (including the authors' own work) in the Introduction and Discussion section is not always fully accurate or sufficiently complete. For example, in discussing the discrepancy between their previous report that neutrophils with hyperlobulated nuclei did not have improved migration compared to cells with banded nuclei (Ginsven et al. J Immunology 2019), the authors suggest that this may be explained by the variable pore size in collagen and the ability of neutrophils to find the path of least resistance. However, this glances over the fact that their previous study also included transwell migration assays with precisely defined 3um diameter pores, in which cells cannot migrate through alternative larger openings. Thus, the authors miss out on a more detailed discussion of potential mechanisms that could explain these differences.

In the first part of the introduction describing the importance of nuclear deformability in cell migration through tight spaces, the authors should include seminal studies such as Wolf et al. JCB 2013, PMID: 23798731; Harada et al. JCB 2014, PMID: 24567359; and Rowat et al. JBC 2013, PMID: 23355469).

The statement that "rupture of the nucleus envelope enables passage through small pores" is intriguing, but although nuclear envelope rupture in response to migration has been described in multiple studies (for example, in the Raab et al. and Denais et al. Science 2016 publications cited later in the text, plus multiple works by the Discher group), this reviewer is not aware of any systematic study showing that nuclear envelope rupture enables migration through small constrictions. The authors should revise the text accordingly.

6) Page 8. In their discussion, the authors seem to suggest that the improved ability of cells to migrate through tight constrictions results from differences in the susceptibility to nuclear envelope rupture, DNA damage, and cell death. However, none of the results presented by the authors here seem to indicate differences in cell death, given that the results are presumably based on the tracking of only living cells, and the time scale of the experiments is likely too short to reflect DNA damage induced difference in cell behavior. Thus, it is not quite clear what point the authors are trying to make. The authors should clarify their discussion accordingly.

© 2023. Published by The Company of Biologists under the terms of the Creative Commons Attribution License (https://creativecommons.org/licenses/by/4.0/).

7) Figure 2. The authors should improve the labeling of Figure 2F to clarify that the different boxes correspond to the matching colors in panel B, which is not obvious from the current description.

First revision

Author response to reviewers' comments

General
We are delighted to read that the reviewers appreciated the novelty of the results and the experimental approach we used to address the outstanding question of whether the lobularity of the neutrophil nucleus facilitates migration through confined spaces. We thank both reviewers for their feedback and have clarified our discussion, analysis, and figures as per the reviewers recommendations and hope that they agree that this manuscript has been strengthened as a result.

Below we answer each of the reviewers' points with reference to additional data or changes made in the manuscript, figures, or supplemental materials. All edits in the revised manuscript are highlighted in red text.

Reviewer 1 Advance Summary and Potential Significance to Field:
The authors used a low level of LPS to induce neutrophils with different maturation stages and numbers of nuclear lobes into circulation. They then isolated the neutrophils, sorted them based on the surface marker, and let them migrate in microfluidic with increasingly narrower spaces. They also stained the nucleus and manually counted the lobs. They discovered a significant correlation between the number of lobes with the neutrophil's ability to migrate in narrow spaces. This is the first report showing this correlation, which is in contrast to previous experiments using other settings. Therefore the results are novel and of interest to the cell migration field.

Reviewer 1 Comments for the Author:
The limitation of the current manuscript is a lack of mechanistic understanding. Does the number of lobes correlate with a softer or more flexible nucleus? In addition, the number of lobes is a direct indication of the maturation stages of neutrophils. The result can be interpreted alternatively that mature neutrophils have an increased ability to migrate through confined spaces. In addition to the morphology of the nucleus, there are significant gene expression changes as neutrophils mature. This is a significant limitation of the current study, and I feel the manuscript is not suitable for publication in JCS.

We thank the reviewer for their comments and agree that investigating mechanism in more detail would be the next step. For the current study, however, our goal was a short-format, proof-of-concept paper for a long-held dogma. We feel that mechanism is beyond the scope of such a short report and would also like to emphasize that it is challenging to do mechanistic studies in primary human neutrophils, given their very short-lived nature in culture and thus the technical challenge of modulating gene expression with tools such as lentiviral vectors or RNA silencing. Indeed, the best way to address this in the future would be probably be by differentiating human induced pluripotent stem cells (iPS) into neutrophils (Trump et al., 2019; PMID: 30793529), which may however also have caveats.

For this short report, we specifically aimed for an experimental design that was as physiologically relevant as possible, taking advantage of a unique human endotoxin administration study. Previous studies have characterized the contribution of nuclear lamina proteins in the passage through transwell pores through modulation in the cell line HL-60, which is commonly used as a neutrophil

© 2023. Published by The Company of Biologists under the terms of the Creative Commons Attribution License (https://creativecommons.org/licenses/by/4.0/).

approximate (Rowat et al., 2013). However, the nuclei of HL-60 cells differ from the nuclei of primary neutrophils. Critically, expression of key nuclear lamina proteins including LBR, Lamin AC, Lamin B1, Lamin B2, Emerin, and LAP2ß are significantly lower, even negligible, in human peripheral blood granulocytes compared to granulocyte-differentiated HL-60 cells (Olins et al., 2008). Thus, we felt it was important to test the hypothesis specifically in primary human neutrophils given the documented differences between these cells and the HL-60 cell line, despite the caveat that using primary neutrophils would limit our ability to investigate molecular mechanism(s).

Aware of these caveats, we designed this study with a two-pronged approach. By performing parallel experiments where neutrophils would be sorted either based on functional subset or assessed strictly by lobularity, we sought to rule out the effect of maturation-related gene-expression changes. Banded neutrophils are indeed thought to be younger than segmented and hypersegmented neutrophils. However, in terms of protein expression banded neutrophils cluster together with the segmented subset and hypersegmented neutrophils form a separate cluster (Tak et al., 2017). If age or protein expression would dominate the effect on migration through narrow pores one would not expect such the gradual effect between the three subsets that we observed. Of note, the most enriched protein clusters that differed between the subsets related to effector functions, RNA binding, and extracellular vesicle formation, rather than migratory or nuclear related processes (Tak et al., 2017). In our current study, we have checked that select proteins of interest do not differ between the subsets, though at this stage we cannot rule out gene expression changes that track with nuclear lobulation and we have now included this as a caveat in the discussion (**revised manuscript, page 8**). Importantly, the fact that the number of lobes showed a stronger relationship with the measured migration parameters than did the functional subsetting of neutrophils is consistent with the idea that nucleus segmentation indeed plays an important role in migration in tight spaces.

In summary, then, this study is the first, to our knowledge, to provide direct experimental evidence that nuclear lobes have a role in facilitating constrained migration in neutrophils. We think that a key strength of our study is that we utilized the naturally occurring heterogeneity in nuclear phenotype among neutrophils mobilized in response to endotoxin administration. However, the use of primary human neutrophils is at the same time a limitation of this study, as these neutrophils cannot easily be manipulated limiting mechanistic experiments. We hope that nonetheless the reviewer will agree with the value of publishing our succinct experimental results for this long-standing question regarding the role of neutrophil nuclear lobes in their migration. We further hope it will lead to follow up studies to address the mechanism queries which, as the reviewer points out, are important.

Reviewer 2 Advance Summary and Potential Significance to Field:
The authors have submitted a manuscript describing the effect of nuclear lobulation on migration through narrow microfluidic channels in heterogeneous populations of primary human neutrophils, demonstrating that neutrophils with more lobes had an improved ability to migrate through the narrow channels than neutrophils with less lobulated nuclei. The manuscript uses ingenious microfluidic networks to study the migration of the cells in confined conditions and relates the migration behavior to various molecular and morphological parameters, identifying nuclear lobulation as a key determinant for confined migration. The movies included in the manuscript are beautiful to watch, and the fact that the authors used primary human fibroblasts and highlight the heterogeneous nature of the cells is considered a major strength.

Reviewer 2 Comments for the Author:
Nonetheless, the manuscript in its current form has several limitations that must be addressed by the authors to better support their interpretation, provide context for the findings, and improve the robustness of the findings.

1) In their presentation of the results and their interpretation, the authors refer to previous work

© 2023. Published by The Company of Biologists under the terms of the Creative Commons Attribution License (https://creativecommons.org/licenses/by/4.0/).

characterizing human neutrophils, particularly their own prior work to describe the association between nuclear lobulation and C16 and CD62L levels (Ginsven et al. J Immunology 2019) and a previous proteomic study (Tak et al. Blood 2017). Since that the authors work with primary human cells, which may be extremely variable, it is important to confirm the results of the prior studies in their current experimental system, especially when correlating results from their current measurements with data from those previous studies to investigate a potential association between protein levels vs. nuclear morphology (Supp. Fig. 1C-D). The authors should provide data for protein measurements in the same cells as used in their experiments, rather than simply assume that the cells used in their studies have similar composition as reported previously. If the data presented in Figure S1C-D is from new measurements, rather than literature data, the authors should clarify this in the manuscript and provide a description of the measurements in the Methods section.

For the results presented in Fig. S1E, the authors should relate the number of nuclear lobes to the levels of CD16 and CD62L, i.e., the classification they used in the current manuscript, and not just as function of the three different categories 'banded', 'mature', and 'hypersegmented'). If these categories are considered to be equivalent, this should be explicitly stated and validated.

To avoid confusion, we have now better clarified the nuclear morphology of the subsets defined by CD16 and CD62L expression by referring to the 'mature' subset as 'segmented' in our manuscript instead. Throughout the study any reference to the 3 categories: banded, segmented and hypersegmented indicates that the neutrophils were sorted by their CD16 and CD62L expression as was done in van Grinsven et al. 2019. Thus, in the revised manuscript, neutrophils are now classified as banded ($CD16^{low}$ $CD62L^{high}$), segmented ($CD16^{high}$ $CD62L^{high}$) or hypersegmented ($CD16^{high}$ $CD62L^{low}$). This has been defined in the text (**revised manuscript, page 5**). We have also validated in our own experimental samples the lobularity of each subset in **Figure S1E**.

The protein measurements of fMLF-R, LMNB1, LMNB2, LBR, and LMNA in Figure S1C-D are indeed from a previous proteomic study (Tak et al., 2017), and not from new measurements produced in the current study. These data are available through ProteomeXchange Consortium via the PRIDE partner repository (data set identifier PXD001674; DOI: 10.6019/PXD001674). We do acknowledge the high variability inherent in human data, however we feel that the assumption of protein composition is acceptable here, as the neutrophils subsets sorted and the endotoxin administration between the two studies is the same. Additionally, the samples were isolated, processed, and sorted with the same methods as the previous study. Unfortunately, it was not possible with the material obtained to repeat the proteomics here.

In general, the levels of lamin A/C are low to negligible in circulating neutrophils. Of the laminar proteins, lamin A/C is thought to contribute the most to nuclear stiffness. Indeed, in the previous proteomic study (Tak et al., 2017), lamin A/C was below the limit of detection. At homeostasis, blood neutrophils are mostly the segmented ($CD16^{high}$ $CD62L^{high}$) population. The banded ($CD16^{low}$ $CD62L^{high}$) and hypersegmented ($CD16^{high}$ $CD62L^{low}$) neutrophil populations only emerge in response to inflammatory stimuli. The endotoxemia trial is not currently ongoing (it was halted during COVID), however we did have access to human blood samples from donors who had just experienced heart failure, a source of sterile inflammation which can also elicit the emergence of the additional neutrophil banded and hypersegmented subsets. When we examined lamin A/C levels in neutrophils from heart failure patients, shown in the figure below, we found that the lamin A/C levels of each of the subsets overlapped considerably, and were substantially lower compared to a nonhematopoietic cell type such as Vero cells.

NOTE: We have removed unpublished data that had been provided for the referees in confidence.

© 2023. Published by The Company of Biologists under the terms of the Creative Commons Attribution License (https://creativecommons.org/licenses/by/4.0/).

2) Although the authors' results convincingly demonstrate that neutrophils with highly lobulated nuclei pass through the channel network more efficiently, the current analysis does not sufficiently address if the effect is specific to the migration through very narrow channels, and if improved chemotaxis by the highly lobulated cells could contribute to the effect. To answer these questions, the authors should calculate migration speed for the different categories of neutrophils as a function of the channel size (i.e., do the highly lobulated cells also show a higher migration efficiency in the larger channels, or only in the narrower channels) and also test whether the same effect is observed in the absence of a chemoattractant or when performing chemotaxis experiments in larger channels that do not require nuclear deformation.

We agree with the reviewers assessment that the ideal analysis would be to follow the migration speed as a function of the channel size. Indeed, this is how we initially envisioned the analysis would be done. When we performed this analysis, we realized that because a fairly high percentage of cells (which varied by lobularity) simply did not make it all the way through to the narrowest sections, that data parsed by channel size therefore had a selection bias, especially in the narrower widths. Hence, we used distance traversed as a proxy for migratory capacity, as in **Figure 1D**. While we did not perform migration experiments in the absence of chemoattractant, we have now included the migration speeds for the widest 6um section, as this section includes all cells and is not subject to the same selection bias. These data indicate that in larger channels that required no nuclear deformation, there was no difference in migration speed between the sorted neutrophil subsets. This additional analysis requested by the reviewer (shown below) has now been included as additional panel of the supplementary figure (**revised Figure S1B**).

NOTE: We have removed unpublished data that had been provided for the referees in confidence.

3) The current description of the migration analysis is difficult to follow due to the confusing terminology and missing definition of terms. For example, the authors use "speed" to describe the rate of movement of the cells along their path (which is consistent with the physical definition of speed), but use "velocity" (which is normally defined as the vector description of the rate and direction of an object's movement) as the directional rate of movement of the cells towards the chemoattractant, if this reviewer's interpretation of the schematic in Figure 1F is correct. Furthermore, the authors refer to "track length, track length, speed, and displacement" and "track duration, length or straightness" in the text, without ever adequately defining these terms. Without a better definition of these terms, the results are very difficult to follow and interpret. The authors should provide a comprehensive and more detailed definition of all the terms used to characterize the cell movement through the microfluidic channels, as these definitions are critical to assess how the nuclear lobulation is association with difference in absolute migration speed, directionality, persistence, chemotaxis, and other parameters of migration.

We apologize if this was confusing. However, the reviewer has indeed correctly interpreted our definitions of both 'speed' and 'velocity'. The speed is the basic calculation of the μm/min the cell moves from frame to frame; accordingly, the average speed is the average value of all the instantaneous speeds per each cell track. The velocity is a vector description of the rate and direction of an objects movement over the course of the entire path of motion. In this case, the object's (the cell's) rate is the speed in μm/min, and the direction of movement is pre-defined as the direction towards the chemoattractant. We have now clarified this better, along with definitions of 'track length', 'track duration', and 'track straightness' in the figure legend and manuscript text (**revised manuscript, page 6,17; and supplementary material page 2,3**).

4) Some figures legends are missing important information on the number of cells used per donor, such as Figures 1E-K. The results presented in panels 1G-I appear to be from a single donor. Were similar results in cells from additional donors?

Figure legends have been updated to reflect the cells per donor in **Fig 1E-K (revised manuscript, page 17)**. These results are representative of all donors analyzed, which is now indicated in the text (**revised manuscript, page 17**).

© 2023. Published by The Company of Biologists under the terms of the Creative Commons Attribution License (https://creativecommons.org/licenses/by/4.0/).

5) The discussion of previous studies (including the authors' own work) in the Introduction and Discussion section is not always fully accurate or sufficiently complete. For example, in discussing the discrepancy between their previous report that neutrophils with hyperlobulated nuclei did not have improved migration compared to cells with banded nuclei (Ginsven et al. J Immunology 2019), the authors suggest that this may be explained by the variable pore size in collagen and the ability of neutrophils to find the path of least resistance. However, this glances over the fact that their previous study also included transwell migration assays with precisely defined 3um diameter pores, in which cells cannot migrate through alternative larger openings. Thus, the authors miss out on a more detailed discussion of potential mechanisms that could explain these differences.

In the first part of the introduction describing the importance of nuclear deformability in cell migration through tight spaces, the authors should include seminal studies such as Wolf et al. JCB 2013, PMID: 23798731; Harada et al. JCB 2014, PMID: 24567359; and Rowat et al. JBC 2013, PMID: 23355469). The statement that "rupture of the nucleus envelope enables passage through small pores" is intriguing, but although nuclear envelope rupture in response to migration has been described in multiple studies (for example, in the Raab et al. and Denais et al. Science 2016 publications cited later in the text, plus multiple works by the Discher group), this reviewer is not aware of any systematic study showing that nuclear envelope rupture enables migration through small constrictions. The authors should revise the text accordingly.

Regarding the discussion of our previous work (van Grinsven et al. 2019), as the reviewer points out, a major caveat with the collagen matrix migration assays was the variable pore sizes. Indeed, in the previous study uniform 3µm pores were also used in the form of transwell chemotaxis assays. In these previous experiments, there was no significant difference in the rate of transmigration, although there was a higher percentage of the hypersegmented neutrophil subset that transmigrated at both the start and end of the experimental readings. The difficulty in interpreting these data is that this increased percentage is present already at the start of the experimental readings, and this assay may fail to capture transmigration which occurs rapidly prior to data collection. In seeming contrast, we found hypersegmented cells were stuck more to the bottom of the transwell membrane than the other subsets. We could not exclude hypersegmented neutrophils had only transmigrated with part of their cell body.

More generally speaking, there were several additional limitations with the use of the transwell chemotaxis assay which we sought to address in the current experimental setup. First, dynamic migration was not tracked in the transwell chemotaxis assay, rather it serves as snapshots of chemotaxis. Additionally, it is not possible to rule out the impact of gravity on the cell passage though the insert, and the design of the transwell assay is a better approximation of diapedesis than of an active 'migration'. The molecular mechanisms underpinning diapedesis differ from those that drive ameboid migration, and here we aim to understand the neutrophils in the context of constrained migration. In this current study, the use of the microfluidics approach allowed us to parse out dynamic readouts on an individual cell basis.

The reviewers point about the nuclear envelope ruptures not directly being tied to enabling migrating through small constrictions is well-taken. Accordingly, we have updated the text (**revised manuscript, page 3**). We regret the omission of a more complete discussion of the aforementioned seminal studies as a result of space constraints and have included them in the updated manuscript (**revised manuscript, page 3-5,8**).

6) Page 8. In their discussion, the authors seem to suggest that the improved ability of cells to migrate through tight constrictions results from differences in the susceptibility to nuclear envelope rupture, DNA damage, and cell death. However, none of the results presented by the authors here seem to indicate differences in cell death, given that the results are presumably based on the tracking of only living cells, and the time scale of the experiments is likely too short to reflect DNA damage induced difference in cell behavior. Thus, it is not quite clear what

© 2023. Published by The Company of Biologists under the terms of the Creative Commons Attribution License (https://creativecommons.org/licenses/by/4.0/).

point the authors are trying to make. The authors should clarify their discussion accordingly.

We agree this discussion point was not well clarified and have removed this.

7) Figure 2. The authors should improve the labeling of Figure 2F to clarify that the different boxes correspond to the matching colors in panel B, which is not obvious from the current description.

We agree with this point and have updated the description in the legend **(revised manuscript, page 19).**

Second decision letter

MS ID#: JOCES/2022/260768

MS TITLE: Nuclear segmentation facilitates neutrophil migration

AUTHORS: Judith N Mandl, Nienke Vrisekoop, Connie Shen, Eva Mulder, Wiebe Buitenwerf, Aron Jansen, Matthjis Kox, and Jeremy Postat
ARTICLE TYPE: Short Report

We have now reached a decision on the above manuscript.

To see the reviewers' reports and a copy of this decision letter, please go to: https://submit-jcs.biologists.org and click on the 'Manuscripts with Decisions' queue in the Author Area. (Corresponding author only has access to reviews.)

As you will see, both reviewers raise a number of substantial criticisms that prevent me from accepting the paper at this stage. While they appreciated the response to their initial critiques, there still remain several issues that need to be resolved. It doesn't appear that new experiments need to be performed but reanalyzing data already in hand should suffice for several points made by both reviewers. I would be pleased to see a revised manuscript.

Please ensure that you clearly highlight all changes made in the revised manuscript. Please avoid using 'Tracked changes' in Word files as these are lost in PDF conversion.

I should be grateful if you would also provide a point-by-point response detailing how you have dealt with the points raised by the reviewers in the 'Response to Reviewers' box. Please attend to all of the reviewers' comments. If you do not agree with any of their criticisms or suggestions please explain clearly why this is so.

Reviewer 1

Advance summary and potential significance to field

I agree with the author that the strength of the manual script is the use of the LPS-induced primary neutrophils and an interesting microfluidic of decreasing width to show different migration abilities of neutrophils with different numbers of lobes. The result is interesting.

Comments for the author

However, the current study does not support the conclusion that neutrophil lobes facilitate neutrophil migration. The data are correlative observations that do not support a causal relationship. The number of lobes and the maturation stage is tightly correlated.

© 2023. Published by The Company of Biologists under the terms of the Creative Commons Attribution License (https://creativecommons.org/licenses/by/4.0/).

Indeed, the Tak 2017 reference repeatedly cited demonstrated that CD62Ldim is a hypersegmentated population with increased nuclear lobes (2-4, mostly 3). This population display increased gene expression in cell adhesion, actin cytoskeleton organization, and response to stimulus pathways, all related to cell chemotaxis in narrow spaces. The conclusion of the paper, cells with 3+ lobes display an increased ability to migrate through narrow channels is consistent with the proteomics data. Therefore, cell maturation and related gene expression can still be the fundamental explanation for the different migration capacities.

The data in Figure 2, especially with the 1-2 lobe, is quite dispersed. What is the rationale for not separating the 1-2 lobe group into groups of 1 lobe and 2 lobes? A cell with 1 lobe is equivalent to a banded neutrophil, and with 2 lobes is equivalent to a segmented neutrophil. This separation is important to support the author's claim that "the number of lobes showed a stronger relationship with the measured migration parameters than did the functional subsetting of neutrophils".

Reviewer 2

Advance summary and potential significance to field

The authors report an interesting findings linking increased nuclear lobulation in neutrophils to an enhanced ability to migrate through small constrictions, building on a growing body of work that highlights the importance of the nucleus in the cells ability to migrate through confined spaces. The use of primary human neutrophils presents a major plus. However, it is current form, the conclusion that the observed enhanced migration is due to increased nuclear lobulation, and not some other confounding factor such as maturity or increased chemotaxis, is not sufficiently supported by the data, reducing the significance of the findings.

Comments for the author

The authors have submitted a revised manuscript that addresses several of the reviewers' comments raised for the original submission. However, there are still a few important items that have not been adequately addressed by the authors.

1) It is still unclear whether the differences in migration through the maze is directly caused by differences in nuclear lobulation, or if nuclear lobulation simply correlates with another parameter that drive the enhanced migration, such as increased maturity or responsiveness to the chemoattractant. The new data presented in Figure S2B showing similar migration speeds ameliorates some of these concerns, but a more careful analysis would nonetheless substantially strengthen the author's interpretation. Ideally, experiments should include conditions in the absence of the chemoattractant, or use of alternative chemoattractants to test if they produce similar results. The authors argue that the stronger effect seen when classifying cells based on nuclear lobulation (Fig. 2C, D, E; Suppl. Fig. 2) compared to classification based on the cell surface receptors into banded, segmented, and hypersegmented cells (Fig. S1B), but given that banded cells have on average only 1-2 nuclear lobes, whereas hypersegmented cells frequently have 3+ lobes (Fig. S1E), wouldn't one expect better agreement between these two analyses? At a minimum, the authors should indicate in the results shown in Fig. 2C, D, and E for each of the data points whether it corresponded to 'banded', 'segmented', or 'hypersegmented' subset of cells, which would indicate whether the classification has an effect independent of nuclear lobulation. In addition, the authors should perform a more rigorous statistical analysis that considers the individual contributions of the effects from the cell surface marker based classification (i.e. banded, segmented, hypersegmented) vs. those from nuclear lobulation. Such an analysis, which should be doable based on the current images and data, could shed further light on which factor is the major contributor to the enhanced migration ability.

2) Although this reviewer recognizes the difficulty of working with human samples, the explanation provided by the authors for not confirming levels of key proteins in the different cell subsets is not sufficient. The authors do not have to perform a full proteomic analysis, but in the spirit of robustness and reproducibility, the authors should confirm the relative levels of key proteins listed in Suppl. Fig. S1C-D in their cells, particularly levels of fMLF-R, which could be

© 2023. Published by The Company of Biologists under the terms of the Creative Commons Attribution License (https://creativecommons.org/licenses/by/4.0/).

directly related to the cells responsiveness to the chemoattractant, and which appears to vary substantially based on the proteomic data. Ideally, this should be perform on a single cell basis to correlate protein levels with nuclear segmentation, but at a minimum should be reported for the three different cell subsets. The data presented on the lamin A/C levels presented in the response to reviewers is somewhat re-assuring, but given that lamin A/C was reported to be absent in all three cell subsets, it is the least informative of the five proteins. Furthermore, in the spirit of transparency, any data presented to the reviewers should also be included in the revised manuscript.

Additional minor comments:

3) In addition, the figure legend for Suppl. Fig. S1C,D should clarify that the data presented in this manuscript are based on a previous study (and should reference this study and the accession information), and not from the cells used in the current study.

4) The author's use and definition of 'velocity' remains problematic, since based on the author's description it is not based on the actual direction of the cell's migrations, but on the pre-defined direction of the gradient. In that case, the authors should refer to it as the velocity towards the gradient, rather than the general velocity.

Second revision

<u>Author response to reviewers' comments</u>

<u>General</u>
We thank the reviewers for the additional feedback provided in response to our revised manuscript. As agreed by the editor in response to our correspondence (see cover letter), we were unfortunately not able to provide additional data requested by the reviewers, as we do not have these available from our completed human study (and neutrophils do not survive freeze-thaws so we cannot bank patient material we can return to). However, we have addressed all remaining minor points by reviewer 2, and added a sentence to the discussion to explicitly state the specific caveat that both reviewers raised and with which we are in agreement with (although currently unable to address experimentally).

<u>Major points</u>
Both reviewers raised the caveat that an as yet unidentified factor that is correlating with nuclear lobularity may explain the differential neutrophil migration behaviour in the constricted channels. As we cannot rule this out, we have added a sentence to state this caveat in our discussion and hope that this is an acceptable compromise (**revised manuscript, page 8**).

<u>Minor points</u>
- *The figure legend for Suppl. Fig. S1C,D should clarify that the data presented in this manuscript are based on a previous study (and should reference this study and the accession information), and not from the cells used in the current study.*

The supplemental figure legend for S1C,D has been updated to indicate the original study and repository that the proteomics derive from (**revised supplemental, page 2**).

- *The author's use and definition of 'velocity' remains problematic, since based on the author's description it is not based on the actual direction of the cell's migrations, but on the pre-defined direction of the gradient. In that case, the authors should refer to it as the velocity towards the gradient, rather than the general velocity.*

We have further clarified that the velocity vector direction is towards the chemokine gradient (**revised manuscript, page 5,7**).

© 2023. Published by The Company of Biologists under the terms of the Creative Commons Attribution License (https://creativecommons.org/licenses/by/4.0/).

Third decision letter

MS ID#: JOCES/2022/260768

MS TITLE: Nuclear segmentation facilitates neutrophil migration

AUTHORS: Judith N Mandl, Nienke Vrisekoop, Connie Shen, Eva Mulder, Wiebe Buitenwerf, Aron Jansen, Matthjis Kox, and Jeremy Postat
ARTICLE TYPE: Short Report

I am happy to tell you that your manuscript has been accepted for publication in Journal of Cell Science, pending standard ethics checks.

© 2023. Published by The Company of Biologists under the terms of the Creative Commons Attribution License (https://creativecommons.org/licenses/by/4.0/).
